# Attention Deficit and Memory Function in Children with Bronchial Asthma: A Systematic Review and Meta-Analysis of 104,975 Patients with Trial Sequential Analysis

**DOI:** 10.3390/children12081013

**Published:** 2025-07-31

**Authors:** Plamen Penchev, Daniela Milanova-Ilieva, Lyubomir Gaydarski, Petar-Preslav Petrov, Kostadin Ketev, Pavel Stanchev, Noor Husain, Nikolai Ramadanov

**Affiliations:** 1 Faculty of Medicine, Medical University of Plovdiv, 4000 Plovdiv, Bulgaria; 2 Department of Pediatrics, St. George’s University Hospital, 4000 Plovdiv, Bulgaria; 3 Department of Anatomy, Histology and Embryology, Medical University-Sofia, 1000 Sofia, Bulgaria; 4 Department of Anatomy, Histology and Embryology, Medical University of Plovdiv, 4000 Plovdiv, Bulgaria; 5 Medical Simulation Training Center, Medical University of Plovdiv, 4000 Plovdiv, Bulgaria; 6 Clinic of Endocrinology and Metabolic Diseases, St. George’s University Hospital, Medical University of Plovdiv, 4000 Plovdiv, Bulgaria; 7 Department of Pharmacology, Indira Gandhi Institute of Medical Sciences, Patna 800014, India; 8 Center of Orthopaedics and Traumatology, Brandenburg Medical School, University Hospital Brandenburg, Brandenburg an der Havel, 14770 Brandenburg, Germany; 9 Faculty of Health Science Brandenburg, Brandenburg Medical School Theodor Fontane, Brandenburg an der Havel, 14770 Brandenburg, Germany

**Keywords:** ADHD, bronchial asthma, memory function, attention deficit, children

## Abstract

**Introduction:** Asthma is a chronic respiratory disease affecting approximately 5 million children in the US, but little is known about whether asthma alters children’s attention and memory functions. Most studies on this topic focus on psychiatric and QoL outcomes rather than cognitive functions, leaving a gap in the literature. We aimed to conduct a systematic review and meta-analysis to evaluate the attention deficit and memory function outcomes in children with bronchial asthma. **Methods:** A systematic search was conducted in PubMed, Web of Science, and Cochrane Library from inception to 28 February 2025 for studies evaluating attention deficit and memory function in children with bronchial asthma. Outcomes of interest included attention deficit and memory function. Statistical analysis was performed with R 4.3.1. Heterogeneity was accessed using the I^2^ statistics and Cochrane Q test. The standardized mean difference (SMD) with restricted maximum-likelihood estimator random-effects method was computed for all outcomes. **Results:** A total of seven studies were included in the final meta-analysis, comprising 104,975 patients, of whom 10,200 (9.7%) had bronchial asthma (mean age ± 8.98 years, mean 45% females). In the pooled analysis, children with asthma had a worsened attention deficit compared to the healthy group (SMD 0.29; 95% CI [0.07; 0.51]; *p* = 0.01; I^2^ = 92%). However, no statistically significant difference was found in memory function between groups (SMD −0.24; 95% CI [−1.81; 1.33]; *p* = 0.77; I^2^ = 96%). **Conclusions:** Children with asthma showed significantly higher attention deficit scores compared to healthy children. No statistically significant differences were observed in memory function between the groups. These findings may have implications for early cognitive screening in pediatric asthma management.

## 1. Introduction

Asthma is one of the most common chronic diseases of childhood and adolescence, with an overall prevalence between 2.1 and 32.2% among 13–14-year-old adolescents and between 4.1 and 32.1% among 6–7-year-old children [1]. Beyond the physical symptoms of the disease, asthma can also result in psychological distress, social difficulties, and academic challenges in affected children [1,2]. A recent meta-analysis discovered that children with asthma were perceived by their parents as having more behavioral problems [3]. These behavioral difficulties were more inward-focused, such as anxiety, depression, and somatic complaints, and outward-directed, including aggressive and delinquent behavior.

Most existing studies are limited by small sample sizes, retrospective study designs, methodological heterogeneity, and a focus on the quality of life, behavioral, social, and mental health problems in children with asthma. Several large-scale studies and meta-analyses have examined associations between asthma and ADHD or atopic diseases using odds ratios, risk ratios, or hazard ratios [4,5,6,7]. However, these studies did not assess cognitive outcomes such as attention or memory using standardized psychometric measures in comparison to healthy controls. Thus, the neurocognitive burden of asthma in children remains insufficiently explored. To date, very little is known about memory function and attention deficit among these patients. Previous studies have shown that children with asthma treated with higher doses of corticosteroids experience reduced verbal memory compared with children receiving lower doses [8]; other studies also reported difficulties in attention, executive function, and memory function [9,10,11,12]. However, the absence of a comparison group of children without asthma in the previously mentioned studies precludes any conclusions about the individual associations between asthma and attention deficit, and asthma and memory function, when compared to healthy children, leaving a knowledge gap in the literature. Previous meta-analyses primarily focused on broader psychiatric comorbidities of asthma, such as ADHD and autism spectrum disorders [13,14,15], but did not evaluate specific cognitive domains such as attention and memory using standardized mean difference (SMD) metrics.

Given the lack of robust evidence, we conducted a systematic review and meta-analysis to evaluate whether children with asthma show significantly different attention deficit and memory function outcomes compared to healthy children. Our study synthesizes data from multiple studies to provide a comprehensive understanding of the cognitive burden associated with asthma. To the best of our knowledge, this is the first meta-analysis evaluating the relationship between asthma, attention deficit, and memory function by comparing children with asthma with healthy children. This study aims to test the hypothesis that children with asthma will exhibit significantly worse attention performance and memory function compared to healthy controls. Specifically, we examined the pairwise associations between (1) asthma and attention deficit, and (2) asthma and memory function, using validated cognitive outcomes. By doing so, this study aims to inform future guidelines and highlight the importance of addressing attention deficit and memory functioning in children with asthma.

## 2. Methods

### 2.1. Eligibility Criteria

This systematic review and meta-analysis followed the Cochrane Handbook for Systematic Reviews of Interventions and the Preferred Reporting Items for Systematic Reviews and Meta-Analysis Statement [16,17]. This meta-analysis did not require Institutional Review Board approval because it used data from previously published and publicly available articles. Restrictions were applied to only English-language, peer-reviewed articles [18]. Gray literature was excluded. Studies that met all the following criteria were included in the meta-analysis: (1) observational studies (case/control, cohort, cross-sectional) or randomized trials; (2) studies with children who have bronchial asthma; (3) studies that report at least one of the following outcomes: attention deficit, memory function assessed using validated measurement tools; and (4) studies that include healthy patients as a control group. Studies were excluded if they met one of the following criteria: (1) did not include children with asthma; (2) no outcomes of interest; (3) different control group; (4) overlapping populations; (5) gray literature, conference abstracts, dissertations, case reports/series, reviews; (6) they reported effect estimates as HR, OR, or RR rather than continuous outcomes suitable for SMD pooling. This systematic review and meta-analysis was registered with the International Prospective Register of Systematic Reviews (PROSPERO) under the ID “CRD420250651281” [18].

### 2.2. Search Strategy and Data Extraction

We systematically searched PubMed, Scopus, and Cochrane Central from inception to 28 February 2025 with the following search strategy: (“children with asthma” OR “asthma in children” OR “asthmatic children” OR “childhood asthma”) AND (“healthy children” OR “non-asthmatic children” OR “comparison group”) [18]. Although databases such as Google Scholar, SpringerLink, Wiley Online Library, Wolters Kluwer, and Taylor & Francis were not directly searched, we chose PubMed, Scopus, and Cochrane Central for their methodological rigor, indexing reliability, and relevance to health sciences literature. Additionally, backward snowballing of references of the included studies was conducted to capture relevant studies beyond the database scope. Two authors (P.P. and K.K.) independently extracted the data using predefined search criteria, quality assessment methods, and Rayyan software (http://new.rayyan.ai, accessed on 28 February 2025) [19]. Any disagreements between these authors were resolved through consensus.

### 2.3. Endpoints and Subgroup Analyses

The meta-analysis included attention deficit and memory function endpoints. Additionally, we conducted a subgroup analysis for each endpoint based on the risk of bias assessment.

### 2.4. Quality Assessment

The risk of bias was assessed using the Cochrane Collaboration’s tool for assessing the risk of bias in non-randomized studies of interventions (ROBINS-I) [20]. The ROBINS-I tool categorizes the risk of bias as low, moderate, serious, or critical. Two authors (P.P. and N.H.) independently performed the assessments, resolving disagreements through consensus. Publication bias was evaluated using traditional and contour-enhanced funnel plots with the trim-and-fill method, which allows better interpretation of asymmetry related to statistical significance thresholds, in line with recommendations by Nakagawa et al. (2017) [21]. Additional methods such as *p*-curve or *p*-uniform analysis were not feasible due to the absence of reported exact *p*-values or test statistics in all included studies. Following the Cochrane guidelines, the Egger test was not performed because fewer than 10 studies were included in the meta-analysis [16].

### 2.5. Statistical Analysis 

The standardized mean difference (SMD) with 95% confidence intervals (CI) was computed to compare effects for continuous endpoints using the restricted maximum-likelihood estimator random-effects method [22,23]. A random-effects model was applied for all outcomes to account for demographic and methodological variability. Heterogeneity was assessed using the I^2^ statistic and the Cochran Q test. Two-sided *p*-values < 0.05 were regarded as statistically significant. Subgroup analyses were performed based on the risk of bias assessment to minimize the risk of selection bias. Leave-one-out (LOO) sensitivity analyses were also conducted to assess the robustness of the findings. A Baujat plot was generated to identify studies contributing most to heterogeneity and their influence on the overall meta-analytic results. This diagnostic tool visually represents the balance between a study’s contribution to heterogeneity (x-axis) and its weight in the meta-analysis (y-axis), aiding in the interpretation of outlier or highly influential studies. Statistical analysis was performed using R software version 4.3.1 with the packages “metafor” and “meta” [18,24]. To address the issue of data non-independence, we ensured that no overlapping datasets were included, and each study contributed unique patient populations. This approach follows the guidance outlined by Nakagawa et al. (2017) to minimize violations of the assumption of independence in meta-analyses [21].

### 2.6. Trial Sequential Analysis (TSA)

To assess the robustness of the meta-analysis and control for type I and type II errors due to sparse data or repeated significance testing, Trial Sequential Analysis (TSA) was performed. TSA was conducted using Trial Sequential Analysis Viewer version 0.9.5.10 Beta with a two-sided significance level of 5% and a power of 80% [18,25]. The required information size (RIS) was calculated based on an anticipated relative risk reduction (RRR) of [18%], a control event rate (CER) of [20%], and heterogeneity correction using a random-effects model. Adjusted cumulative Z-curves were plotted to evaluate whether the results reached the TSA monitoring boundaries for statistical significance or the RIS.

## 3. Results

### 3.1. Study Selection and Baseline Characteristics 

**Figure 1:**
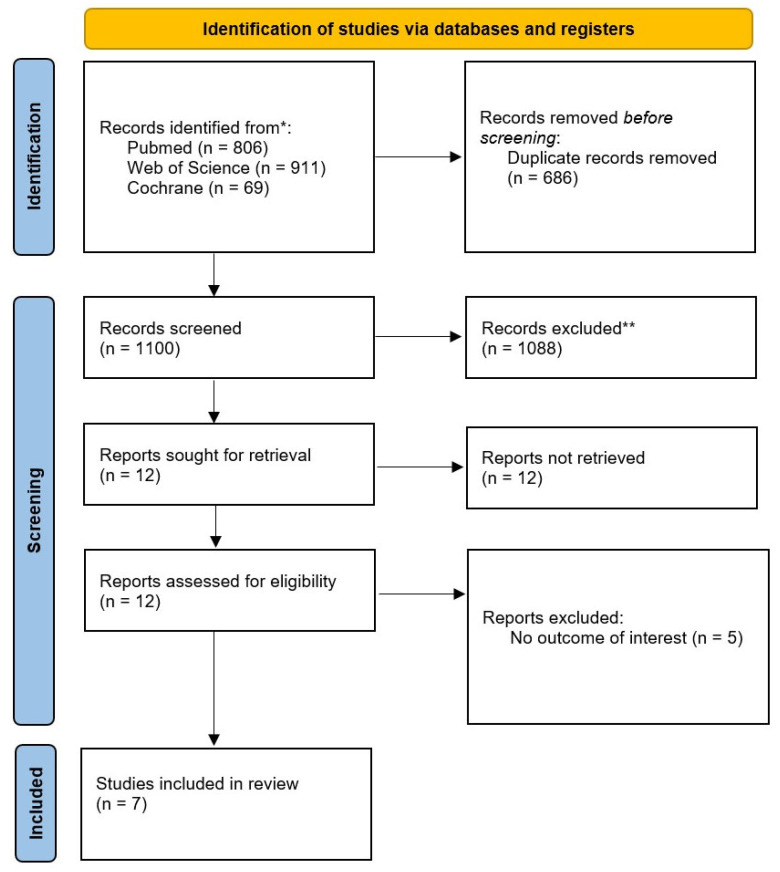
PRISMA flow diagram illustrating the process of study identification, screening, eligibility assessment, and final inclusion. The diagram outlines the number of records identified through database searching, duplicates removed, records excluded at the title/abstract level, full-text articles assessed for eligibility, and reasons for exclusion. Ultimately, seven studies met all inclusion criteria and were included in the final meta-analysis.

The search strategy yielded a total of 1786 results. After removing duplicate records and unrelated articles or abstracts, the remaining 12 studies were fully reviewed to determine whether they met the inclusion and exclusion criteria (Figure 1). Seven studies were included, with 104,975 patients [1,2,9,12,26,27,28]. Of these, 10,200 patients (9.72%) had asthma and were included in our analyses. The mean age of the population was 8.98 ± SD years. The females accounted for a mean of 45%. Population characteristics are presented in Table 1.

### 3.2. Pooled Analyses of All Included Studies

#### 3.2.1. Attention Deficit

**Figure 2:**
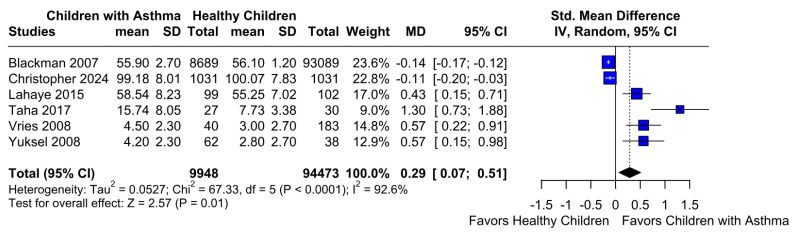
Forest plot of standardized mean differences (SMDs) for attention deficit between children with asthma and healthy controls. Each horizontal line represents an individual study’s SMD and 95% confidence interval (CI), while the diamond at the bottom indicates the pooled effect estimate. A positive SMD indicates greater attention deficit in the asthma group. The analysis revealed a statistically significant difference favoring higher attention deficit in children with asthma (SMD = 0.29; 95% CI [0.07, 0.51]; *p* = 0.01; I^2^ = 92%) [1,2,9,26,27,28].

**Figure 3:**
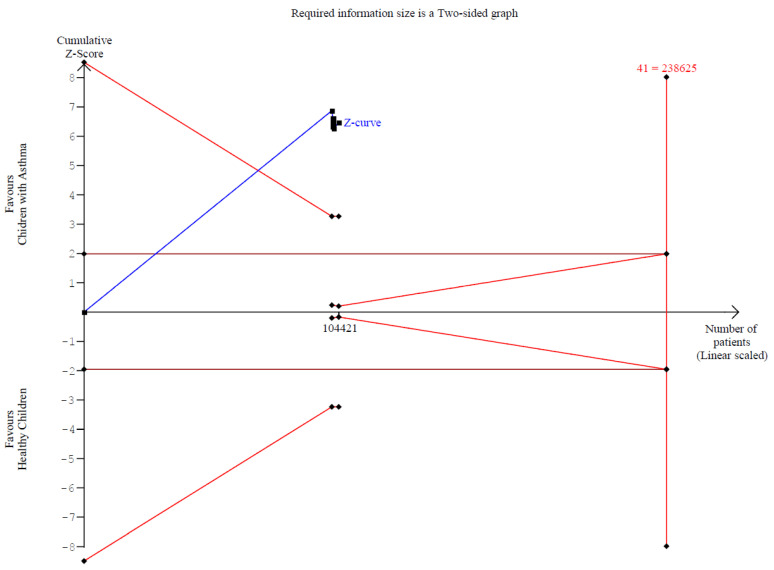
Trial Sequential Analysis (TSA) for attention deficit comparing children with asthma to healthy controls. The cumulative Z-curve (blue line) crosses the conventional significance boundary and the trial sequential monitoring boundary, indicating that the pooled evidence is sufficient to confirm a statistically significant difference in attention deficit without risk of random error. The required information size (RIS) is represented by the vertical red line, and the monitoring boundaries are plotted in red. This analysis confirms the robustness of the finding that children with asthma exhibit higher attention deficit than healthy peers.

**Figure 4:**
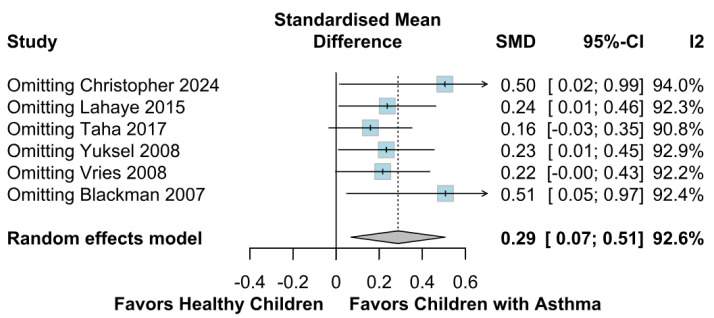
LOO sensitivity analysis for attention deficit. This analysis assesses the robustness of the pooled effect by sequentially removing one study at a time and recalculating the overall SMD. The plot demonstrates that the overall effect size for attention deficit remains consistent across all iterations, with no single study unduly influencing the result. Although the association remains statistically significant throughout, heterogeneity remains high across all iterations, reflecting methodological and population differences among included studies [1,2,9,26,27,28].

**Figure 5:**
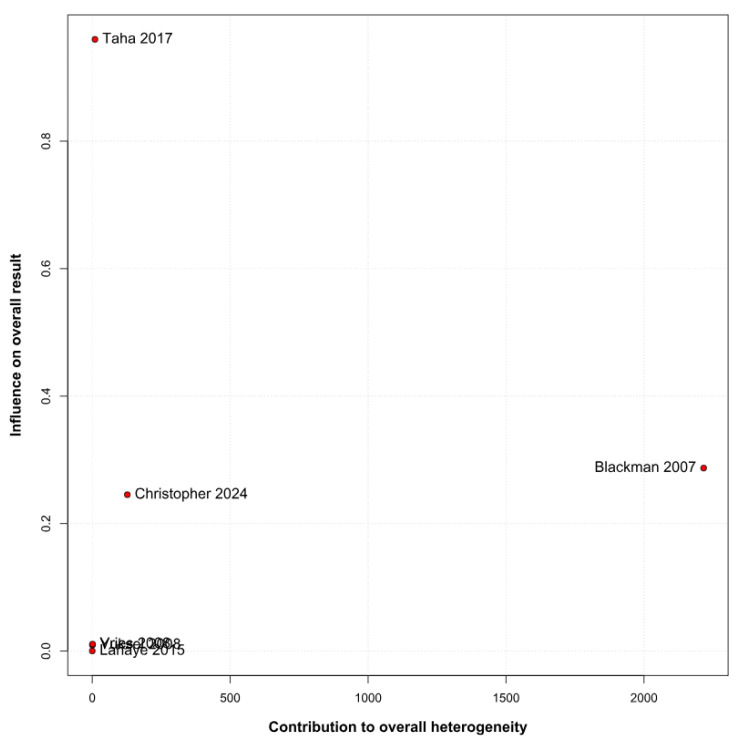
Baujat plot for attention deficit assessing the influence of individual studies on overall heterogeneity and pooled effect size. Each point represents a single study, with its position determined by its contribution to the total heterogeneity (*x*-axis) and its influence on the overall meta-analytic result (*y*-axis). Studies positioned in the upper-right quadrant contribute both high heterogeneity and strong influence. The plot identifies Taha et al. (2017) as a potentially influential outlier, indicating a need for cautious interpretation of its contribution [1,2,9,26,27,28].

A significantly worse attention deficit was observed in children with asthma (SMD 0.29; 95% CI [0.07; 0.51]; *p* = 0.01; I^2^ = 92%) (Figure 2). This indicates substantial heterogeneity, likely due to differences in study designs, populations, and assessment tools. TSA confirmed statistically significant differences between the groups (Figure 3). A LOO analysis was performed to test the robustness of our results. The overall effect size remained consistent across all iterations and the result remained significant in all cases (SMD 0.29 [0.07; 0.51]; 95% CI; *p* = 0.01; I^2^ = 92%) (Figure 4). This suggests that no single study has a disproportional influence on the overall outcome. The Baujat plot identified in the study by Taha et al. [26] is potentially influential, contributing substantially to the overall result (Figure 5). Details of the scales and tools used to assess attention deficit across the included studies are summarized in Table 2.

#### 3.2.2. Memory Function

**Figure 6:**
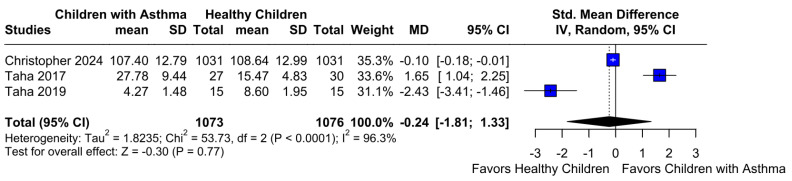
Forest plot illustrating the comparison of memory function between different groups. Each horizontal line represents the effect size and 95% confidence interval (CI) for individual studies included in the meta-analysis [9,12,26]. The overall pooled effect size is shown as a diamond at the bottom of the plot. The plot indicates no statistically significant difference in memory function between the groups, as the confidence intervals cross the line of no effect. This suggests comparable memory performance across the analyzed cohorts.

**Figure 7:**
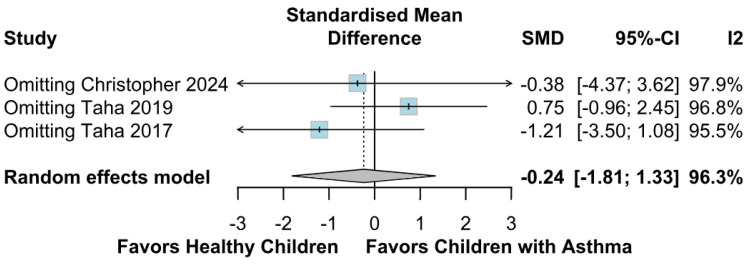
LOO sensitivity analysis for memory function effect size. This plot shows the overall effect size calculated by sequentially omitting one study at a time from the meta-analysis [9,12,26]. The consistency of the effect size across all iterations indicates that no single study disproportionately influenced the results. In all cases, the effect remained non-significant, confirming the robustness of the finding that there is no statistically significant difference in memory function between groups. The horizontal axis represents the effect size, and the vertical dashed line indicates the null effect.

**Figure 8:**
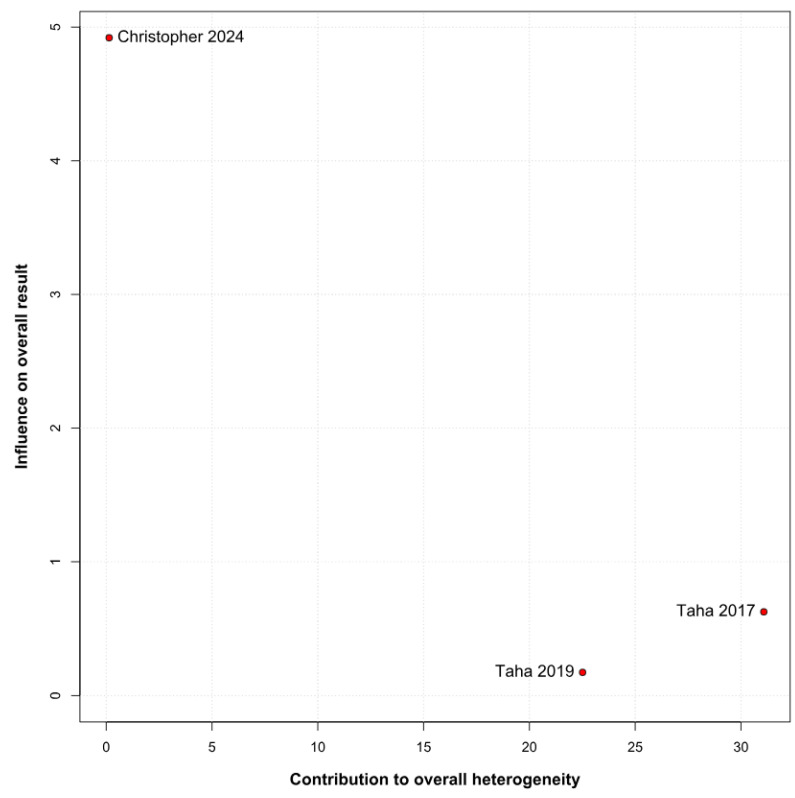
Baujat plot depicting the contribution of individual studies to overall heterogeneity and their influence on the meta-analysis of memory function [9,12,26]. Each point represents a study, plotted according to its influence on the overall effect size (*x*-axis) and its contribution to the heterogeneity statistic (Q, *y*-axis). Studies located toward the upper-right corner contribute most to heterogeneity and have the greatest impact on the pooled results. The plot identifies Christopher et al. (2024) as a potentially influential outlier, indicating a need for cautious interpretation of its contribution [9].

No statistically significant difference between the groups was observed (SMD −0.24; 95% CI [−1.81; 1.33]; *p* = 0.77; I^2^ = 96%) (Figure 6). This indicates substantial heterogeneity, likely due to differences in study designs, populations, and assessment tools. A LOO analysis was performed to test the robustness of our results. The overall effect size remained consistent across all iterations. The result remained non-significant in all cases (SMD −0.24 [−1.81; 1.33]; 95% CI; *p* = 0.77; I^2^ = 96%) (Figure 7). This suggests that no single study has a disproportional influence on the overall outcome. The Baujat plot identified in the study by Christopher et al. [9] is potentially influential, contributing substantially to the overall result (Figure 8). Details of the scales and tools used to assess memory function across the included studies are summarized in Table 3.

### 3.3. Subgroup Analyses

#### 3.3.1. Attention Deficit Based on the Risk of Bias

**Figure 9:**
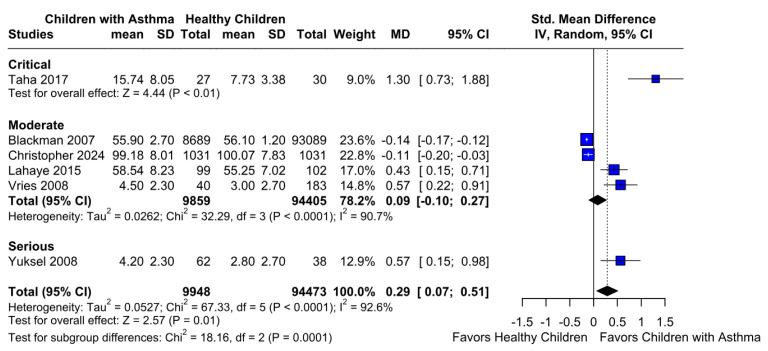
Forest plot showing the subgroup analysis of attention deficit based on risk of bias in included studies [1,2,9,26,27,28]. The plot displays the effect sizes and 95% confidence intervals (CIs) for each study, grouped according to their risk of bias classification (e.g., moderate, critical, serious). The overall pooled effect indicates a statistically significant difference in attention deficit between groups. The horizontal lines represent individual study CIs, and the diamond reflects the combined effect size within each subgroup. This subgroup analysis highlights how study quality may influence the observed differences in attention deficit outcomes.

A statistically significant difference between the groups was found (SMD 0.29 [0.07; 0.51]; 95% CI; *p* = 0.0001; I^2^ = 92%) (Figure 9).

#### 3.3.2. Memory Function Based on the Risk of Bias

**Figure 10:**
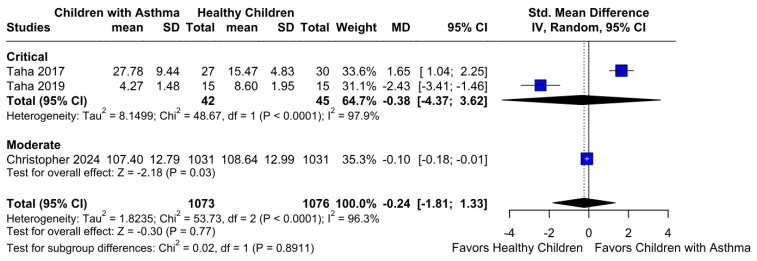
Forest plot representing subgroup analysis of memory function according to risk of bias categories in the included studies [9,12,26]. Each study’s effect size and 95% confidence interval (CI) are displayed, grouped by their assessed risk of bias (e.g., moderate, critical). The overall pooled effect size indicates no statistically significant difference in memory function between groups across all risk of bias subgroups. This analysis assesses whether study quality influences the observed effects on memory function.

No statistically significant difference between the groups was observed (SMD −0.24 [−1.81; 1.33]; 95% CI; *p* = 0.8911; I^2^ = 96%) (Figure 10).

### 3.4. Quality Assessment

**Figure 11:**
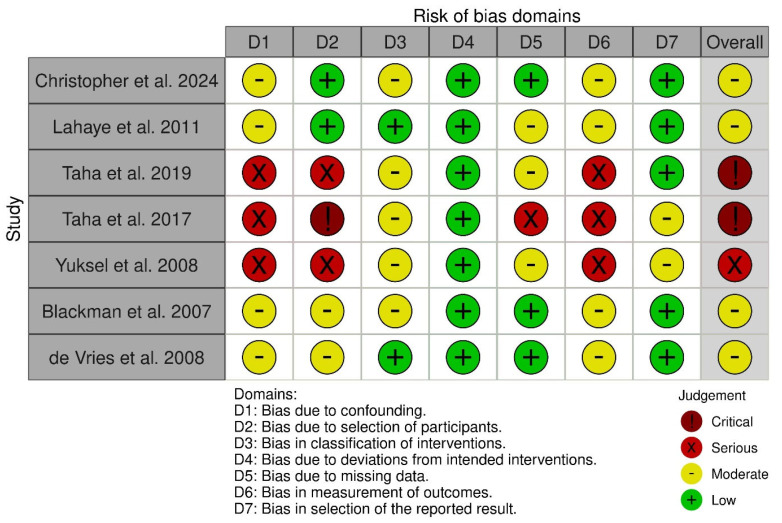
Risk of bias assessment for the studies included in the meta-analysis [1,2,9,12,26,27,28]. This figure summarizes the evaluation of potential biases across multiple domains, such as selection bias, confounding bias, classification bias, measurement of outcomes bias, and reporting bias, for each study. The assessment helps determine the overall quality and reliability of the evidence by highlighting areas of low, moderate, serious, or critical risk of bias, which may affect the validity of the meta-analytic findings.

**Figure 12:**
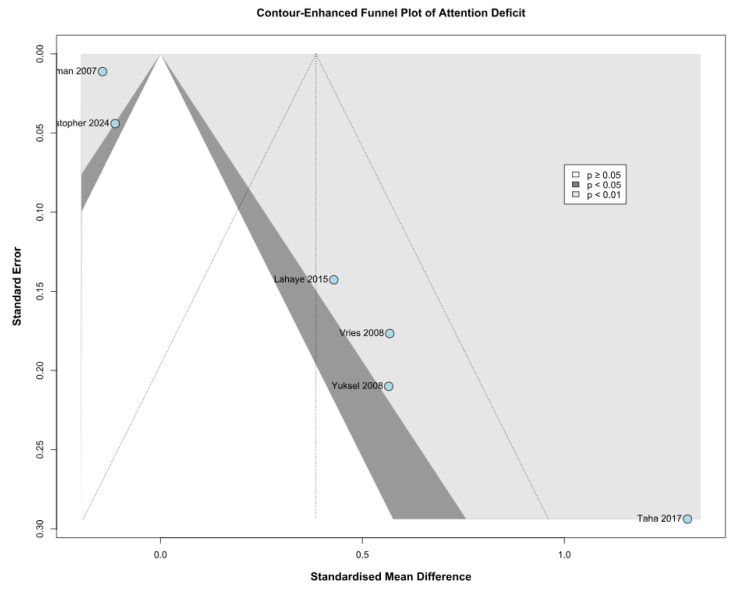
Contour-enhanced funnel plot evaluating publication bias in studies assessing attention deficit [1,2,9,26,27,28]. Each point represents an individual study, plotted according to its effect size (point estimate) on the *x*-axis and study precision (weight or standard error) on the *y*-axis. The shaded contours indicate regions of statistical significance, helping distinguish between potential publication bias and other causes of asymmetry. The trim-and-fill method was applied to adjust for suspected missing studies, estimating their influence on the overall effect size and assessing the robustness of the meta-analytic findings.

**Figure 13:**
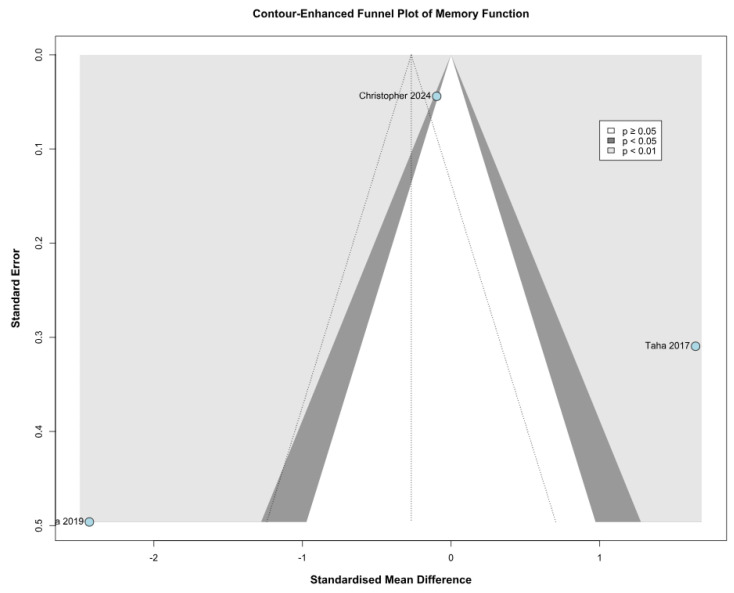
Contour-enhanced funnel plot assessing publication bias in studies evaluating memory function ([9,26,27]). Individual studies are plotted according to their effect sizes (point estimates) on the x-axis and their precision (study weights or standard errors) on the *y*-axis. The shaded contours represent areas of statistical significance, which help to differentiate between publication bias and other sources of asymmetry. The trim-and-fill method was employed to identify and adjust for potentially missing studies, providing a corrected estimate of the overall effect size and evaluating the robustness of the meta-analytic results.

Among the seven included studies, four were assessed as having a moderate risk of bias, one as having a serious risk of bias, and two as having a critical risk of bias based on the ROBINS-I tool. The evaluation of the studies is reported in Figure 11. The most common sources of bias were confounding and selection bias, with four studies judged to have a moderate risk of bias and three having a serious risk of bias in these domains. In the funnel plot analysis, asymmetry was observed, indicating a probable presence of publication bias (Figure 12 and Figure 13).

## 4. Discussion

In this systematic review and meta-analysis of seven studies and 104,975 patients, we compared the attention deficit and memory function of children with asthma and healthy children. The main findings from the pooled analyses were as follows: (1) children with asthma may be at increased risk of attention deficit compared to healthy children; (2) in terms of memory function, there was no statistically significant difference between children with asthma and the control group. Our review highlights the lack of comprehensive data on the attention deficit and memory function outcomes of children with asthma compared to healthy children. This meta-analysis provides a more in-depth evaluation of cognitive outcomes, specifically attention deficit and memory function, revealing both neutral and adverse effects. These findings enhance our understanding of the nuanced relationship between asthma and its broader cognitive impacts and complement those of previous meta-analyses [13,14,15], which examined associations between asthma and psychiatric disorders but did not focus specifically on cognitive metrics such as memory and attention as continuous outcomes.

This selective deficit in attention aligns with several studies that have examined cognitive and behavioral outcomes in pediatric populations [1,2,9,12,26,27,28]. The high heterogeneity observed (I^2^ > 90%) reflects the methodological and demographic diversity among studies, including variability in psychometric instruments and patient populations. For instance, research suggests that children with asthma face challenges related to physical health and exhibit higher rates of ADHD, depression, anxiety, and learning disabilities, with asthma severity directly correlating with the prevalence of these cognitive deficits [27]. These children also experience significantly more school absenteeism—half of those with severe asthma miss at least 10 days per year—and face more significant academic challenges, as indicated by increased parental contact regarding school performance and a nearly threefold higher likelihood of grade repetition [9,27]. According to Yuksel et al. [2], asthmatic children were found to have significantly higher attention deficit scores compared to their non-asthmatic peers, underscoring the broader impact of chronic respiratory conditions on neurodevelopment. These observations are consistent with our results on the attention deficit outcome, suggesting that both direct physiological factors and secondary influences such as socioeconomic adversity and chronic disease burden may drive the cognitive consequences of such conditions.

Moreover, while deficits in immediate and working memory have been reported in individual studies—such as those comparing verbal and visual memory performance [12]—the overall meta-analytic result indicates that memory function remains largely unaffected when aggregating data across multiple investigations. However, the high heterogeneity observed (I^2^ > 90%) reflects the methodological and demographic diversity among studies, including variability in psychometric instruments and patient populations. The included studies assessed a variety of memory domains, such as immediate recall, verbal learning, and visual memory, which may not be directly comparable. For instance, while Taha et al. [12] evaluated working memory and verbal learning, Christopher-Hayes et al. [9] used a visual episodic memory test, potentially contributing to divergent results across studies. This discrepancy could reflect methodological differences between studies, including variability in memory assessment tools or the heterogeneity of the sample characteristics. For example, some studies have shown that while immediate short-term verbal memory and working memory are impaired [12], the capacity for verbal learning remains relatively intact, suggesting that the cognitive impact of the condition may be domain-specific. These findings suggest that the observed deficits in immediate and working memory and visual memory may underlie the broader cognitive challenges identified in the affected group, while the capacity for verbal learning remains relatively intact [12]. In contrast to our cognitive focus, several prior studies have explored the broader epidemiological association between asthma and ADHD. Miyazaki et al. [4] conducted a meta-analysis showing an increased ADHD risk among children with allergic diseases. Chang et al. [5] examined atopic disease risk among siblings of ADHD patients, while Park et al. [6] investigated the bidirectional relationship between ADHD and asthma over time. Zhuoran et al. [7] further applied Mendelian randomization to support a shared genetic predisposition. While informative, these studies reported outcomes as odds ratios or hazard ratios and did not evaluate attention or memory using direct psychometric tools. Our meta-analysis adds to this body of literature by examining specific cognitive domains in a clinical comparison between asthmatic and healthy children. Christopher et al. [9] further revealed that children with a history of asthma performed worse on episodic memory, processing speed, inhibition, and attention tasks compared to matched controls. These cognitive deficits persisted even after adjusting for parental income and racial distribution covariates. The findings suggest that early-onset asthma may have a lasting negative impact on cognitive development, particularly in episodic memory, emphasizing the need for further research into the underlying mechanisms linking asthma to neurodevelopmental outcomes [9].

Additional evidence from the literature further supports these differential cognitive outcomes. Longitudinal and cross-sectional analyses have shown that early-onset asthma may negatively influence episodic memory and other cognitive domains, although processing speed, inhibition, and attention have been more consistently affected [9]. Similarly, investigations into the executive function of asthmatic children reveal global deficits—particularly in cognitive flexibility—that may underlie both learning difficulties and attentional impairments [26]. One potential explanation for the observed executive function deficit might be due to early childhood asthma-related hypoxia affecting frontal lobe development [2,29,30]. These studies highlight the complex nature of this disease, demonstrating that its clinical manifestations—such as varying degrees of asthma severity and its association with socioeconomic factors (as noted by Blackman et al. [27])—can differ significantly across individuals [9,26,27]. However, despite this variability, one common finding is the consistent disruption in attentional processes among affected individuals [1,2,9]. This suggests that, regardless of differences in external factors like disease severity or socioeconomic status, attentional deficits may serve as a central cognitive hallmark of the condition, potentially indicating a shared underlying mechanism that warrants further investigation.

Furthermore, behavioral studies comparing groups of children receiving different types of asthma management (e.g., inhaled corticosteroids versus otolaryngological care) have reported that children on specific medication regimens exhibit poorer behavioral functioning and higher ADHD symptom scores [28]. Such findings emphasize the potential role of the underlying pathology and its treatment in shaping cognitive outcomes, thereby offering a possible explanation for the significant attention deficit observed in the meta-analysis. In contrast, psychosocial assessments have generally not found a parallel impairment in memory function, corroborating the meta-analytic evidence that memory remains relatively preserved despite attentional deficits.

Our meta-analysis reinforces the presence of a modest yet statistically significant attention deficit in children with bronchial asthma while revealing considerable heterogeneity that likely arises from differences in study design, population characteristics, and assessment methods. When these findings are integrated with the broader literature on chronic pediatric conditions such as asthma, it becomes clear that attentional processes are particularly vulnerable—potentially due to direct neurobiological impacts and indirect influences from socioeconomic factors and treatment regimens [1,2,9,27]. In contrast, the relative preservation of memory function suggests that these cognitive domains may be less affected by the condition or that current measurement approaches might not be sensitive enough to detect subtler impairments [9,12]. These insights underscore the importance of developing targeted interventions to address attention deficits and highlight the need for further research into the mechanisms underlying these cognitive disparities.

This meta-analysis has limitations. First, there is considerable heterogeneity among the included studies, which likely reflects differences in study design, population characteristics, and assessment methods; this variability hinders the interpretation of our pooled effect estimates. Second, although sensitivity analyses confirmed the robustness of the attention deficit finding, the influence of one study (Taha et al.) suggests that our overall result may be sensitive to individual study effects [12]. Third, the lack of significant differences in memory function could be due to methodological inconsistencies in how memory was assessed across studies, limiting our ability to draw definitive conclusions about this cognitive domain. Moreover, the included studies did not allow for stratification based on asthma treatment modalities, particularly the use of corticosteroids or other confounding factors such as socioeconomic status, or comorbidities, which may independently affect cognitive outcomes such as memory and attention. This represents a significant limitation in our ability to isolate the effects of asthma itself.

Additionally, while we did not search other large databases (e.g., Google Scholar, SpringerLink), we selected PubMed, Scopus, and Cochrane for their rigorous indexing standards and relevance. Backward reference screening was performed to minimize the risk of missing pertinent studies. We acknowledge that our search strategy was limited to three major databases and could be improved in future studies by including additional sources such as Embase, PsycINFO, and CINAHL to enhance comprehensiveness and reduce the risk of missing relevant studies.

Several potentially relevant studies, including those by Miyazaki et al. [4], Chang et al. [5], Park et al. [6], and Zhuoran et al. [7], were excluded for not meeting our inclusion criteria—specifically, the absence of a healthy control group, reporting effect estimates in incompatible formats (e.g., OR, HR, RR), or study design mismatch. For example, Miyazaki et al. was a meta-analysis, which was excluded a priori based on our criteria restricting inclusion to original observational or interventional studies. Additionally, many of these studies focused on risk associations or comorbidity rather than directly measuring attention or memory function using standardized psychometric tools. We acknowledge their contribution to the broader literature and recognize that their findings may complement our results in future mixed-method or umbrella network meta-analytic designs.

While we used contour-enhanced funnel plots with the trim-and-fill method to assess publication bias, some bias assessment tools (e.g., *p*-curve analysis or Egger’s test) could not be performed due to data reporting limitations across the included studies. The studies included in this review used diverse psychometric instruments to assess attention deficit and memory function, introducing heterogeneity that may influence pooled results. These tools are detailed in Table 2 and Table 3. We tried to address these concerns through LOO and Baujat plot sensitivity analyses, TSA, and subgroup analyses based on the risk of bias.

## 5. Conclusions

This meta-analysis of 104,975 patients suggested that children with asthma showed significantly higher attention deficit scores compared to healthy controls. However, no statistically significant differences were found between groups regarding memory function. These findings underscore the need for early cognitive screening in asthmatic children, particularly in school and primary care settings, to enable timely support and intervention. Further studies with larger sample sizes and longer follow-up periods are needed to explore potential underlying mechanisms.

## Figures and Tables

**Table 1:** Baseline characteristics of the included studies.

Study	No. Patients	Children with Asthma	Healthy Children	Age *	Female, %	Race, Caucasian, %	Severe Asthma, %	High SES, %
Blackman, 2007 [27]	101,778	8689	93,089	7.8	66%	8.1	5	8.8
Christopher-Hayes, 2024 [9]	474	237	237	9.9	49	62	N/A	N/A
	2062	1031	1031	11.9	43	54		
Lahaye, 2011 [1]	201	99	102	11.4	34	N/A	54	61
Taha, 2019 [12]	30	15	15	4.7	60	N/A	N/A	N/A
Taha, 2017 [26]	57	27	30	12.5	41	N/A	N/A	N/A
Vries, 2008 [28]	273	40	233	4.4	27	N/A	N/A	8
Yuksel, 2008 [2]	100	62	38	9.2 ± 1.5	40	N/A	N/A	N/A

* Mean age, SES—socioeconomic status.

**Table 2:** Summary of studies assessing attention deficit in children with asthma using ADHD-related behavioral scales.

Study	Psychometric Tool for ADHD/Attention Deficit
Blackman, 2007 [27]	Parent-reported diagnosis (NSCH single-item question)
Christopher-Hayes, 2024 [9]	NIH Toolbox—Flanker Inhibitory Control and Attention Test
Lahaye, 2011 [1]	CBCL—Attention Problems Subscale (parent-reported)
Taha, 2017 [26]	WCST—Sustained attention and memory (via non-perseverative errors)
Vries, 2008 [28]	DSM-IV-based questionnaire (covering attention deficit, hyperactivity, impulsivity); CBCL (attention axis)
Yuksel, 2008 [2]	Conners’ Parent Rating Scale-48 (CPRS-48)

**Table 3:** Summary of studies assessing attention deficit in children with asthma using memory function behavioral scales.

Study	Psychometric Tool for Memory Function
Christopher-Hayes, 2024 [9]	NIH Toolbox—Picture Sequence Memory Test
Taha, 2017 [26]	WCST—Sustained attention and memory (via non-perseverative errors)
Taha, 2019 [12]	Digit Span (forward), Backward Digit Span, Adapted Rey Auditory Verbal Learning Test (RAVLT), MVPT-3, RCFT

## Data Availability

All data analyzed in this study were obtained from previously published articles available in public databases (PubMed, Scopus, and Cochrane Central). The full list of included studies is available in the References section.

## References

[1] Lahaye M., Luminet O., Van Broeck N., Bodart E. (2012). Psychological, social and school implications of asthma: A comparison of Belgian French-speaking children having asthma with healthy children. Acta Clin. Belg..

[2] Yuksel H., Sogut A., Yilmaz O. (2008). Attention deficit and hyperactivity symptoms in children with asthma. J. Asthma.

[3] McQuaid E.L., Kopel S.J., Nassau J.H. (2001). Behavioral adjustment in children with asthma: A meta-analysis. J. Dev. Behav. Pediatr..

[4] Miyazaki C., Koyama M., Ota E., Swa T., Mlunde L.B., Amiya R.M., Tachibana Y., Yamamoto-Hanada K., Mori R. (2017). Allergic diseases in children with attention deficit hyperactivity disorder: A systematic review and meta-analysis. BMC Psychiatry.

[5] Chang T.H., Tai Y.H., Dai Y.X., Chang Y.T., Chen T.J., Chen M.H. (2019). Risk of Atopic Diseases among Siblings of Patients with Attention-Deficit Hyperactivity Disorder: A Nationwide Population-Based Cohort Study. Int. Arch. Allergy Immunol..

[6] Park H.J., Kim Y.H., Na D.Y., Jeong S.W., Lee M.G., Lee J.H., Yang Y.N., Kang M.G., Yeom S.W., Kim J.S. (2023). Long-term bidirectional association between asthma and attention deficit hyperactivity disorder: A big data cohort study. Front. Psychiatry.

[7] Zhuoran Z., Xiaoman W., Rui Y., Qingjiu C. (2025). Association Between ADHD and Pediatric Asthma: Results from a Large-Sample Cross-Sectional Study of National Surveys and Mendelian Randomization Analyses. J. Atten. Disord..

[8] Bender B.G., Lerner J.A., Kollasch E. (1988). Mood and memory changes in asthmatic children receiving corticosteroids. J. Am. Acad. Child Adolesc. Psychiatry.

[9] Christopher-Hayes N.J., Haynes S.C., Kenyon N.J., Merchant V.D., Schweitzer J.B., Ghetti S. (2024). Asthma and Memory Function in Children. JAMA Netw. Open.

[10] Hajek C.A., Yeates K.O., Anderson V., Mackay M., Greenham M., Gomes A., Lo W. (2014). Cognitive outcomes following arterial ischemic stroke in infants and children. J. Child Neurol..

[11] Jiang K., Zhu L., Ge Y., Shangguan Q., Chen Y., He Y., Wu J., Qin C., Xiong J., Zhao J. (2021). Brain network study of attentional cognitive impairment in children with bronchial asthma. Res. Sq..

[12] Taha H., Khalil M. (2019). Verbal and visual memory performances of children with moderate-into-severe asthma. Pol. Psychol. Bull..

[13] Schans J.V., Çiçek R., de Vries T.W., Hak E., Hoekstra P.J. (2017). Association of atopic diseases and attention-deficit/hyperactivity disorder: A systematic review and meta-analyses. Neurosci. Biobehav. Rev..

[14] Kaas T.H., Vinding R.K., Stokholm J., Bønnelykke K., Bisgaard H., Chawes B.L. (2021). Association between childhood asthma and attention deficit hyperactivity or autism spectrum disorders: A systematic review with meta-analysis. Clin. Exp. Allergy.

[15] Cortese S., Sun S., Zhang J., Sharma E., Chang Z., Kuja-Halkola R., Almqvist C., Larsson H., Faraone S.V. (2018). Association between attention deficit hyperactivity disorder and asthma: A systematic review and meta-analysis and a Swedish population-based study. Lancet Psychiatry.

[16] Higgins J., Thomas J., Chandler J., Cumpston M., Li T., Page M.J., Welch V.A. (2023). Cochrane Handbook for Systematic Reviews of Interventions Version 6.4 (Updated August 2023).

[17] Page M.J., McKenzie J.E., Bossuyt P.M., Boutron I., Hoffmann T.C., Mulrow C.D., Shamseer L., Tetzlaff J.M., Akl E.A., Brennan S.E. (2021). The PRISMA 2020 statement: An updated guideline for reporting systematic reviews. BMJ.

[18] Penchev P., Ivanov K., Milanova-Ilieva D., Gaydarski L., Kostov K., Boyadzhiev N., Petrov P.-P., Mehandzhiev P., Hyusein R., Velchev V. (2025). Mental Health and Quality of Life in Patients with Untreated Unruptured Intracranial Aneurysms: A Systematic Review and Meta-Analysis of 417,152 Patients with Trial Sequential Analysis. Brain Sci..

[19] Ouzzani M., Hammady H., Fedorowicz Z., Elmagarmid A. (2016). Rayyan—A web and mobile app for systematic reviews. Syst. Rev..

[20] Sterne J.A., Hernán M.A., Reeves B.C., Savović J., Berkman N.D., Viswanathan M., Henry D., Altman D.G., Ansari M.T., Boutron I. (2016). ROBINS-I: A tool for assessing risk of bias in non-randomised studies of interventions. BMJ.

[21] Nakagawa S., Noble D.W.A., Senior A.M., Lagisz M. (2017). Meta-evaluation of meta-analysis: Ten appraisal questions for biologists. BMC Biol..

[22] Hartung J., Knapp G. (2001). On tests of the overall treatment effect in meta-analysis with normally distributed responses. Stat. Med..

[23] Knapp G., Hartung J. (2003). Improved tests for a random effects meta-regression with a single covariate. Stat. Med..

[24] R Core Team (2024). R: A Language and Environment for Statistical Computing. R Foundation for Statistical Computing.

[25] (2021). Trial Sequential Analysis (TSA) [Computer program] Version 0.9.5.10 Beta.

[26] Taha H. (2017). Poor Executive Functions among Children with Moderate-into-Severe Asthma: Evidence from WCST Performance. Front. Psychol..

[27] Blackman J.A., Gurka M.J. (2007). Developmental and behavioral comorbidities of asthma in children. J. Dev. Behav. Pediatr..

[28] de Vries T.W., van Roon E.N., Duiverman E.J. (2008). Inhaled corticosteroids do not affect behaviour. Acta Paediatr..

[29] Fryt J., Pilecka W., Smoleñ T. (2013). Importance of symptom control: Self-regulation in children with diabetes type 1 and asthma. Stud. Psychol..

[30] Fuster J.M. (2002). Frontal lobe and cognitive development. J. Neurocytol..

